# Screening for obstructive sleep apnoea in the general population in Iceland and uptake of positive airway pressure treatment: a prospective cohort study

**DOI:** 10.1136/bmjopen-2026-119621

**Published:** 2026-09-02

**Authors:** Elin Helga Thorarinsdottir, Bryndis Benediktsdottir, Thor Aspelund, Hjordis Sigrun Palsdottir, Sesselja Hreggvidsdottir, Tryggvi Leo Gudmundsson, Ali Reza Matin, Bjorg Eysteinsdottir, Christer Janson, Eva Lindberg, James Potts, Andre FS Amaral, Thorarinn Gislason

**Affiliations:** 1 Faculty of Medicine, University of Iceland, Reykjavik, Iceland; 2 Primary Health Care of the Capital Area, Reykjavik, Iceland; 3 Icelandic Heart Association, Kopavogur, Iceland; 4 Department of Sleep, Landspitali, The National University Hospital of Iceland, Reykjavik, Iceland; 5 Department of Medical Sciences: Respiratory, Allergy and Sleep Research, Uppsala Universitet, Uppsala, Sweden; 6 National Heart and Lung Institute, Imperial College London, London, UK; 7 NIHR Imperial Biomedical Research Centre, London, UK

**Keywords:** PREVENTIVE MEDICINE, Primary Health Care, PUBLIC HEALTH

## Abstract

**Abstract:**

**Objectives:**

To estimate uptake of obstructive sleep apnoea (OSA) screening and initiation and long-term use of positive airway pressure (PAP) treatment in a middle-aged and older general population cohort.

**Design:**

Prospective population-based cohort study.

**Setting:**

Icelandic arm of the multinational Burden of Obstructive Lung Disease follow-up study II (2019–2021)

**Participants:**

378 non-institutionalised adults aged 54–91 years were invited from a general population cohort. 26 with prior OSA diagnosis were excluded. Of the remaining participants, 334 (88.4%) completed a technically adequate home sleep apnoea test.

**Interventions:**

Those with an apnoea–hypopnoea index (AHI)≥15 events/hour were invited for clinical evaluation and when appropriate, referred for PAP treatment. Adherence was assessed 2 years after PAP initiation.

**Main outcome measures:**

Primary outcomes were screening uptake and PAP initiation. Secondary outcomes included long-term PAP use and objective adherence 2 years after referral.

**Results:**

AHI≥15 was identified in 132/334 participants (39.5%). Of these, 123 (93.2%) attended clinical evaluation and 99 (75.0%) were referred for PAP treatment. Of those not referred, six declined PAP and the remainder were advised alternative management or reassessment. At 2-year follow-up, 53/99 (53.5%) were long-term PAP users, 43/99 (43.4%) had discontinued and 3/99 (3.0%) never initiated PAP. Objective adherence data were available for 47/53 long-term users; 21/47 (44.7%) met adherence criteria (≥4 hours/night on ≥70% of nights in the preceding 30 days).

**Conclusions:**

Most adults accepted OSA screening when offered. Among those with moderate-to-severe OSA identified through population screening, most accepted evaluation and PAP treatment, with approximately half becoming long-term users. These findings suggest that population-based detection of OSA followed by routine clinical management is feasible. Future studies should identify subgroups most likely to benefit and evaluate alternative treatment strategies.

**STRENGTH AND LIMITATIONS OF THIS STUDY**

- This study was embedded within a well-characterised, population-based cohort with standardised recruitment and data collection, enhancing generalisability to middle-aged and older adults in the community.
- Obstructive sleep apnoea was assessed using home sleep apnoea testing scored by a single experienced technologist according to contemporary American Academy of Sleep Medicine criteria, ensuring consistency of outcome assessment.
- The use of type 3 sleep studies without electroencephalography may have led to underestimation of sleep-disordered breathing severity compared with full polysomnography.
- Referral for positive airway pressure (PAP) treatment was based on clinical judgement in addition to apnoea–hypopnoea index, which reflects usual practice, but introduces potential variability in treatment eligibility.
- PAP treatment was delivered through routine clinical care pathways, improving real-world relevance but limiting experimental control over treatment decisions and follow-up.

## Introduction

 Obstructive sleep apnoea (OSA) is a highly prevalent and multifactorial sleep disorder associated with substantial cardiovascular, metabolic, neurocognitive and respiratory morbidity.^1^ OSA is also associated with increased perioperative risk, particularly when undiagnosed.^2^ Beyond its clinical burden, OSA carries major economic and safety implications, including increased accident risk, reduced productivity and higher healthcare utilisation, with untreated disease estimated to cost billions annually.^3^

OSA affects an estimated one billion people globally.^4^ Despite its high prevalence and substantial consequences, OSA remains markedly underdiagnosed in primary care settings, with studies showing that most patients at high risk for OSA are not assessed or referred for evaluation, and fewer than one-third of patients with sleep-related symptoms have these documented in their medical records.^5^ Home sleep apnoea testing (HSAT) is recommended for diagnosis of symptomatic adults, where the apnoea–hypopnea index (AHI) is used to assess disease severity.^6^ However, individuals with similar AHI values may differ markedly in symptoms and comorbidities, highlighting the heterogeneity of OSA.^7^ OSA is more common in men and AHI increases with age, although associations with cardiovascular diseases appear to weaken at older ages^8^ and adherence to treatment tends to decline in the very elderly.^9^ Positive airway pressure (PAP) remains the most widely used treatment for OSA and has been shown to improve symptoms, quality of life, blood pressure and safety outcomes.^10^

Given the public health consequences of untreated OSA, there is growing interest in evaluating population-based diagnostic and treatment pathways. This study aimed to assess the feasibility of identifying OSA among middle-aged and older adults through population-based screening with HSAT, and to evaluate subsequent treatment uptake and long-term adherence among those diagnosed.

## Materials and methods

### Study population

The study population is comprised of Icelandic participants in the multinational Burden of Obstructive Lung Disease follow-up study (BOLD II).^11^ The BOLD study is a population-based epidemiological cohort designed to assess the prevalence and risk factors of obstructive lung disease worldwide. In the baseline study (BOLD I, conducted in 2012 in Iceland), non-institutionalised adults aged ≥40 years were randomly sampled from the general population residing in defined catchment areas with at least 150 000 inhabitants.

BOLD II (conducted in 2019–2021 in Iceland) represents a 10-year longitudinal follow-up of the original cohort. The BOLD II study period partially overlapped with the COVID-19 pandemic in Iceland and public health restrictions were in place from March 2020 to February 2022. All surviving participants of the original Icelandic cohort were invited to participate in BOLD II. In addition to the protocol used across all sites of the BOLD II study, Icelandic participants were invited to undergo HSAT and, when indicated, a subsequent clinical evaluation for OSA and consideration of PAP treatment. Individuals referred for PAP treatment were invited for follow-up assessment 2 years after referral.

For this present analysis, inclusion was restricted to BOLD II participants who completed the core questionnaire, reported no prior diagnosis of OSA and had a technically acceptable HSAT study. Participants who had undergone recent HSAT (<1 year) without evidence of OSA were not rescreened and were not included in the analysis. Participants with missing, incomplete or technically inadequate HSAT data were also excluded.

### Data collection

Participants completed standardised interviewer-administered questionnaires and underwent clinical measurements according to the BOLD II protocol.^11^

### Questionnaires and clinical variables

Questionnaires included information on sociodemographic factors (education, employment, household assets), lifestyle exposures (tobacco and cannabis use, occupational and environmental exposures) and general health. Cardiometabolic comorbidities were defined based on self-report of a physician diagnosis of hypertension, diabetes, cardiovascular disease, stroke or OSA.^11^

Height and weight were measured using standardised procedures, and body mass index (BMI) was calculated as kg/m². All study staff completed centralised training prior to data collection.

### Sleep-related symptoms and sleepiness

As part of the core BOLD II questionnaire, participants reported whether they had been told they snored during the past month (yes/no). Daytime sleepiness was assessed by the question “Do you feel very sleepy during the day?” with responses on a five-point Likert scale (never to always). Participants responding ‘frequently’ or ‘always’ were classified as ‘feeling sleepy’. The Epworth Sleepiness Scale (ESS) was used, and a score >10 was considered to indicate increased risk of dozing.

### HSAT

All Icelandic BOLD II participants were invited to undergo HSAT using a type 3 portable monitoring device (T3, Nox Medical, Reykjavik, Iceland). Recorded signals included nasal airflow (pressure cannula), thoracic and abdominal respiratory effort (inductive plethysmography belts), pulse oximetry (Nonin Inc, Plymouth, Minnesota, USA), body position, activity and audio. Studies were scored by a single experienced sleep technologist blinded to clinical data. Recordings were considered technically adequate if they included at least 4 hours of scorable oxygen saturation data and at least two of the following respiratory signals of acceptable quality: nasal airflow, thoracic effort or abdominal effort. Respiratory events were scored according to the 2012 American Academy of Sleep Medicine criteria,^12^ defining hypopnoea as a ≥30% reduction in airflow lasting ≥10 s accompanied by ≥3% oxygen desaturation. The AHI was calculated as the number of events per hour of analysed recording time. The oxygen desaturation index (ODI) was calculated as the number of oxygen desaturation events ≥3% per hour of analysed recording time.

### PAP treatment

All participants received written information regarding their HSAT results. Those with an AHI ≥15 events/hour were invited for a 1-hour clinical evaluation at Landspitali University Hospital. During this visit, general health, OSA-related symptoms and comorbidities were assessed, and the potential benefits of PAP therapy were discussed. Referral for PAP treatment was based on HSAT findings in conjunction with clinical judgement.

Participants with AHI 5–14.9 events/hour were not routinely referred for PAP therapy. They were informed of their HSAT findings and advised to seek further evaluation and possibly some management through their general practitioner. A summary of the study findings, including HSAT results, was also sent to each participant’s general practitioner to support further clinical assessment and management as appropriate.

Participants referred for treatment were managed by the Sleep Department at Landspitali, the sole provider of PAP therapy in Iceland. PAP treatment followed routine clinical care pathways, identical to those offered to patients outside the study. PAP users in Iceland pay a monthly fee of approximately US$ 20 (US$ 4 for older adults and individuals receiving disability benefits). All participants underwent standardised mask fitting and education (30–45 min) by trained staff, with no restrictions on mask type.

Auto-adjusting PAP devices (AutoSet S10, ResMed, Australia) with heated humidification were prescribed, with pressure settings of 6–16 cm H_2_O. Devices were equipped with wireless data transmission enabling remote monitoring of usage and mask leak via a secure cloud-based platform (AirView) for the first year of treatment. Participants who discontinued PAP were instructed to return the device and were no longer charged for its use. During parts of the study period, in-person follow-up was limited due to the COVID-19 pandemic, and patient contact was only conducted remotely.

### Follow-up and PAP adherence

Two years after PAP referral, participants were contacted by letter and telephone and invited for follow-up. They were asked about current PAP use and invited to attend a repeat clinical evaluation, during which PAP devices were brought to the hospital and usage data downloaded. Long-term PAP use was defined as self-reported continued use at the 2-year follow-up. Adherent use was defined as device use for ≥4 hours per night on ≥70% of nights during the preceding 30 days, based on objective device data.

### Patient and public involvement

Patients and members of the public were not involved in the design, conduct, reporting or dissemination plans of this research.

### Statistical analysis

Categorical variables are presented as counts and percentages and were compared using the χ² test. Continuous variables are presented as means±SD and compared using t-tests or ANOVA. Logistic regression analysis was performed to evaluate factors associated with AHI≥15. Multivariable logistic regression models were adjusted for age, sex, BMI, ESS score and snoring. Similarly, logistic regression analyses were performed to evaluate factors associated with long-term PAP use, and multivariable models were adjusted for age and sex. Results from logistic regression analyses are presented as ORs with 95% CIs. A p value<0.05 was considered statistically significant. To assess the potential impact of the COVID-19 pandemic, participants were categorised according to whether they were assessed before or during the period of COVID-19-related public health restrictions in Iceland (from 16 March 2020 to the end of February 2022). Comparisons between groups were performed for key outcomes, including screening participation and PAP adherence. All analyses were conducted in Stata V.19 (StataCorp, College Station, Texas, USA).

This study is reported in accordance with the Strengthening the Reporting of Observational Studies in Epidemiology (STROBE) guidelines.

## Results

### Characteristics of the study population

Among the 757 participants in the BOLD I study in Iceland who had answered the core questionnaire and had a useable spirometry, there were 187 who had died between baseline and follow-up, and additionally 73 who had left the catchment area, were untraceable or could not be contacted and therefore ineligible for participation in the BOLD II study. Non-responders were 119 who actively refused to participate or provided only partial data. Altogether 378 (55% males) completed the core questionnaire in BOLD II (76% cooperation rate).^11^

Compared with BOLD I participants who did not participate in BOLD II, responders were younger (mean age 51.7 vs 61.0 years, p<0.001) but there were no significant differences in BMI (27.9 vs 28.0 kg/m², p=0.73) or frequency of habitual snoring (45.0% vs 47.1%, p=0.57). Responders had slightly higher ESS score (6.7 vs 5.3, p<0.001), although the absolute difference was modest. The proportion with risk of dozing (ESS score>10) was also higher among responders, but this difference did not reach statistical significance (57.8% vs 42.2%, p=0.061).

**Figure 1:**
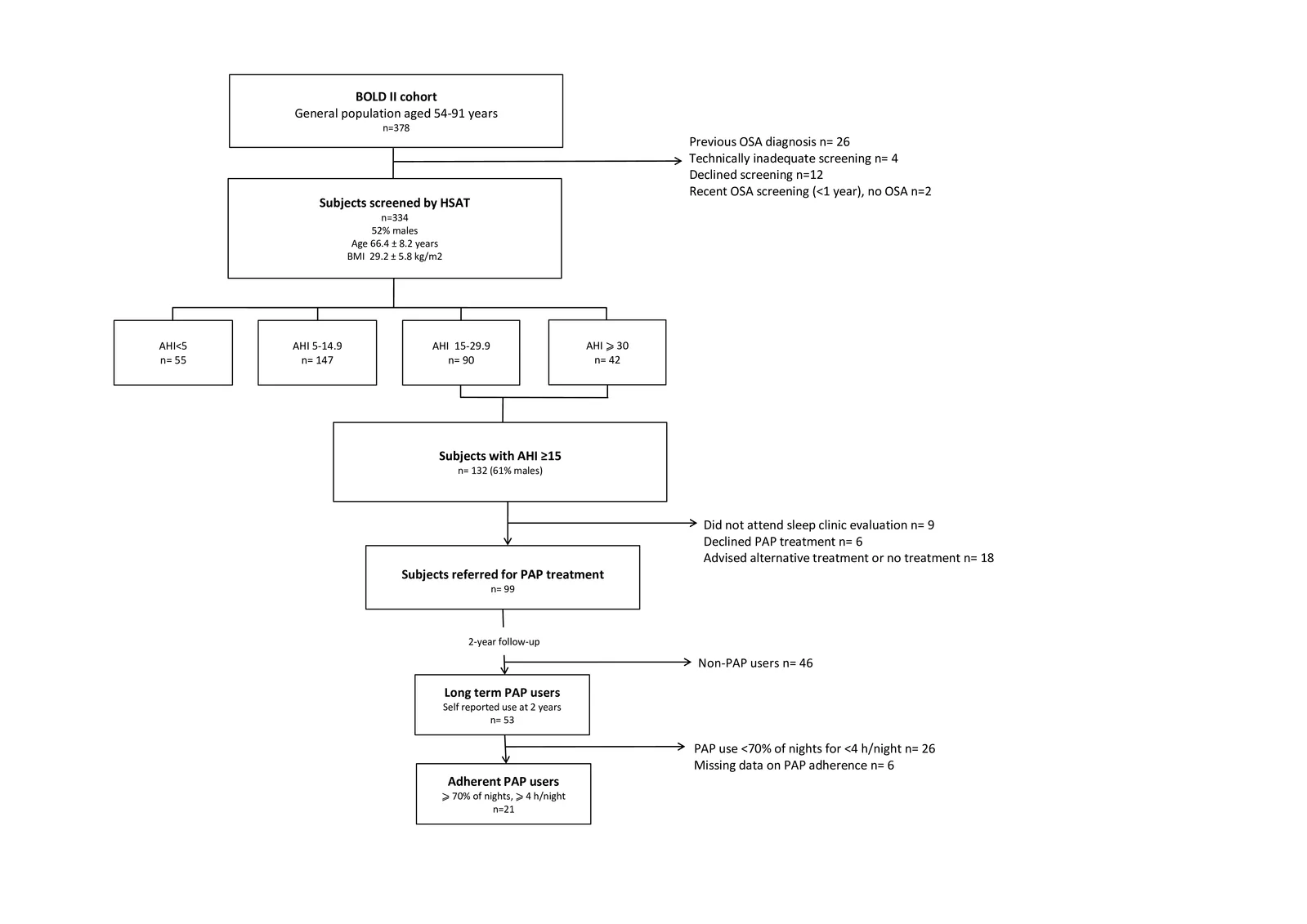
Flow chart of the study population. AHI, apnoea–hypopnoea index; BMI, body mass index; BOLD, Burden of Obstructive Lung Disease; HSAT, home sleep apnoea testing; OSA, obstructive sleep apnoea; PAP, positive airway pressure.

A flow chart of the study population is shown in figure 1. Of the 378 individuals participating in the BOLD II study, 26 (7.2%) reported a previous diagnosis of OSA. An additional two participants had undergone recent OSA screening (<1 year) with no evidence of OSA and were therefore not re-screened. 12 participants declined screening. In total, 35 HSAT studies were initially technically inadequate for various reasons. All but four participants accepted repeat testing and subsequently provided an acceptable HSAT. Participation in screening was significantly lower during the COVID-19 restriction period compared with the pre-pandemic period (90.8% vs 97.1%, p=0.012).

Overall, 334 technically adequate HSAT recordings were available for analysis (173 males (52%), 161 females (48%)). Their mean age was 66.4±8.2 years (range 54–91 years), and mean BMI was 29.2±5.8 kg/m² (figure 1).

### Results of HSAT

#### AHI and demographics

Among the 334 participants with available HSAT studies, 55 subjects (16.4%) had AHI<5, 147 (44.0%) had AHI 5–14.9, 90 (27.0%) had AHI 15–29.9 and 42 (12.6%) had AHI ⩾30 (figure 1). Increasing AHI severity was associated with higher age, a higher proportion of males and higher BMI (table 1). On average, AHI was significantly higher among males than females (AHI 17.7±13.2 vs 13.3±11.6, p=0.001). Obesity (BMI>30) became progressively more common with rising AHI, reaching more than two-thirds in the most severe group (AHI⩾30, table 1). In linear regression, AHI increased by 0.25 events/hour for each additional year of age (β=0.25, p=0.003) and by 0.71 events/hour for an 1 kg/m^2^ increase in BMI (β=0.71, p<0.001). Educational level differed by AHI severity (p=0.003), with a higher proportion of participants with AHI ⩾30 reporting only primary or secondary education and fewer with university education (19.1% in AHI ⩾30 vs 58.2% in AHI<5) (table 1).

**Table 1:** Demographics, general health and OSA symptoms by AHI category

	AHI<5	AHI 5–14.9	AHI 15–29.9	AHI ≥30	p Value*
Subjects, n (%)	55 (16.4)	147 (44.0)	90 (27.0)	42 (12.6)	
Demographics					
Male gender, n (%)	16 (29.1)	77 (52.4)	53 (58.9)	27 (64.3)	**0.001**
Age, years±SD	63.6±7.4	66.2±7.5	67.5±8.4	68.9±9.7	**0.007**
Mean BMI, kg/m^2^±SD	26.9±7.0	27.9±4.4	30.9±6.4	32.7±4.6	**<0.001**
BMI>30 kg/m^2^, n (%)	9 (16.4)	37 (25.2)	47 (52.2)	29 (69.1)	**<0.001**
Smoking, n (%)					0.784
Current	3 (5.5)	7 (4.8)	4 (4.4)	0 (0.0)	
Former	28 (50.9)	80 (54.4)	52 (57.8)	22 (52.4)	
Never	24 (43.6)	60 (40.8)	34 (37.8)	20 (47.6)	
Highest level of education, n (%)					**0.003**
None	0	1 (0.7)	0	0	
Primary school	1 (1.8)	1 (0.7)	2 (2.2)	3 (7.1)	
Lower secondary school	7 (12.7)	29 (19.7)	10 (11.1)	7 (16.7)	
Upper secondary school	7 (12.7)	20 (13.6)	10 (11.1)	5 (11.9)	
Vocational or trade education	8 (14.6)	45 (30.6)	41 (45.6)	19 (45.2)	
University	32 (58.2)	51 (34.7)	27 (30.0)	8 (19.1)	
General health					
Hypertension, n (%)	17 (30.9)	71 (48.3)	43 (47.8)	25 (59.5)	**0.037**
Diabetes, n (%)	2 (3.6)	10 (6.8)	13 (14.4)	7 (16.7)	**0.038**
Cardiovascular disease, n (%)	5 (9.1)	39 (26.5)	22 (24.4)	11 (26.2)	0.061
Stroke, n (%)	2 (3.6)	3 (2.0)	1 (1.1)	2 (4.8)	0.561
OSA symptoms					
Snoring†, n (%)	25 (45.5)	95 (64.6)	70 (77.8)	32 (76.2)	**<0.001**
Feeling sleepy‡, n (%)	8 (14.8)	28 (19.2)	16 (18.2)	8 (19.1)	0.912
ESS score, mean±SD	7.1±4.6	6.2±3.8	6.7±4.2	7.0±4.2	0.400
ESS score>10, n (%)	13 (25.0)	24 (16.3)	14 (15.9)	6 (15.4)	0.486

* p Values were calculated using χ² tests for categorical variables and one-way analysis of variance for continuous variables, comparing AHI categories. Statistically significant results (p<0.05) are shown in bold.

† Yes vs no.

‡ Frequent or always vs never, rarely or sometimes.

OSA, obstructive sleep apnoea; AHI, apnoea–hypopnoea index; BMI, body mass index; ESS, Epworth Sleepiness Scale.

#### AHI categories, co-morbidities and reported OSA symptoms

Reported hypertension was more common in higher AHI categories (30.9% in AHI<5 vs 59.5% in AHI≥30, p=0.037, table 1), and diabetes showed a similar trend (3.6% vs 16.7%, p=0.038). Cardiovascular disease tended to be more prevalent with higher AHI, though this did not reach statistical significance (p=0.061) while reported stroke prevalence did not differ across groups (table 1).

Of classical OSA symptoms, self-reported snoring was significantly different between AHI categories, where those with AHI<5 less often reported snoring compared with those in higher AHI categories (table 1). Subjective sleepiness (mean ESS score, ESS score>10 and feeling sleepy) did not differ significantly across AHI groups (table 1).

In logistic regression analyses, increasing age, male sex, obesity and self-reported snoring were associated with moderate OSA (AHI≥15) (online supplemental table S1). In the multivariable model adjusted for age, sex, BMI category, ESS score and snoring, age, male sex, obesity (BMI≥30 kg/m²) and snoring remained independently associated with moderate OSA, while ESS score>10 was not associated with AHI≥15. Participants with obesity had more than sixfold increased odds of moderate OSA compared with participants with BMI<25 kg/m² (adjusted OR 6.43, 95% CI 2.77 to 14.88) (online supplemental table S1).

### PAP referrals and adherence

Among the 132 subjects with an AHI ⩾15 altogether 99 were referred for PAP treatment after clinical evaluation (figure 1). Nine participants did not attend the post-screening clinical assessment. After clinical interview, only six declined PAP therapy. In addition, 14 participants (all with moderate OSA) were advised alternative treatment, including positional therapy (n=11), mandibular advancement device (n=2) or referral to an ear, nose and throat specialist (n=1). For a further four participants, a mutual decision was made to defer PAP treatment due to significant comorbid health conditions, with advice to seek reassessment should symptoms worsen.

Compared with those not referred, participants referred for PAP therapy significantly more often had a BMI>30 (60.6% vs 37.5%, p=0.041) (online supplemental table S2). Those referred also had significantly more severe OSA (AHI 30.1±12.5 vs 20.0±4.4 events/hour, p<0.001) (online supplemental table S2). Among the 99 referred for PAP treatment three never showed up, 11 returned their PAP device within 3 months and additionally 29 within the first year. During the 2-year follow-up period, two subjects passed away. At the 2-year follow-up, 46 were non-users and 53 subjects were long-term PAP users (figure 1). Of the 53 PAP users, altogether 47 came to the hospital for new evaluation and PAP usage data was downloaded. Of them, 21 (44.7%) were adherent PAP users (using their PAP device≥70% of nights for≥4 hours/night for the past 30 days according to objective data).

Of the 99 participants referred for PAP treatment, only 6 initiated PAP therapy before the COVID-19 pandemic (before 16 March 2020). Among small group who started treatment before the pandemic, 83.3% were PAP users at the 2-year follow-up, compared with 53.3% among those who initiated therapy during the pandemic, although this difference did not reach statistical significance (p=0.152).

#### Factors associated with long-term PAP use and adherence

**Figure 2:**
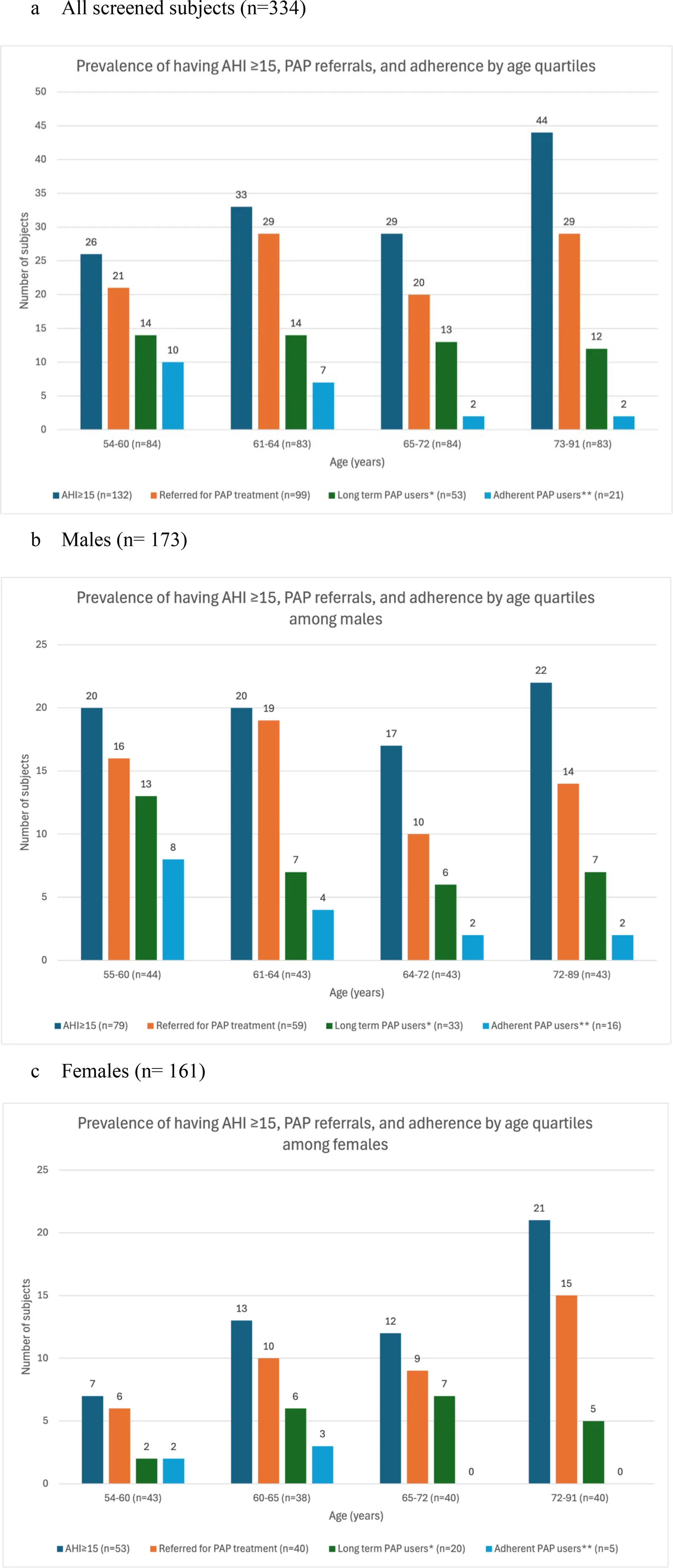
Prevalence of having AHI ≥15, PAP referrals and adherence by age for (A) all screened subjects, (B) males and (C) females. Dark blue, AHI≥15 on screening; orange, referred for PAP treatment; green, long-term PAP users; light blue, adherent PAP users. *Using PAP at the 2-year follow-up, ** Using PAP ≥70% of nights and ≥4 hours per night for the last 30 days. AHI, apnoea–hypopnoea index; PAP, positive airway pressure.

As shown in table 2, adherent PAP users were significantly younger than non-PAP users at the 2-year follow-up (61.5±5.8 vs 68.1±8.3 years, respectively, p=0.002). Of the 167 subjects belonging to the younger half of our sample (54–64 years), 59 (35%) had AHI ⩾15, 50 (30%) got referred for PAP treatment, 28 (17%) became long-term PAP users and 17 (10%) fulfilled criteria for adherent PAP users (figure 2A). The comparative numbers for the 167 in the oldest half of the study population were 73 (44%) had AHI ≥15 and 49 (29%) got referred for PAP treatment, 25 (15%) became long-term users but only 4 (2%) fulfilled criteria for adherent PAP user at the 2-year follow-up. Furthermore, among the 24 women aged>65 years referred for PAP treatment, none were adherent PAP users at the 2-year follow-up (figure 2C). Corresponding results for men are shown in figure 2B.

**Table 2:** Baseline characteristics of participants by PAP compliance and adherence

	Non-PAP users	Long-term PAP users*	Adherent PAP users†
Subjects, n	46	53	21
Demographics			
Male gender, n (%)	26 (56.5)	33 (62.3)	16 (76.2)
Age, years±SD	68.1±8.3	66.4±8.8	**61.5±5.8‡**
Mean BMI, kg/m^2^±SD	30.4±4.9	32.3±5.1	31.5±3.9
BMI>30 kg/m^2^, n (%)	23 (50.0)	**37** (**69.8**)§	14 (66.7)
Smoking, n (%)			
Current	2 (4.4)	1 (1.9)	1 (4.8)
Former	24 (52.2)	33 (62.3)	13 (61.9)
Never	20 (43.5)	19 (35.9)	7 (33.3)
Highest level of education, n (%)			
None	0 (0.0)	0 (0.0)	0 (0.0)
Primary school	0 (0.0)	4 (7.6)	0 (0.0)
Lower secondary school	5 (10.9)	8 (15.1)	4 (19.1)
Upper secondary school	5 (10.9)	7 (13.2)	3 (14.3)
Vocational or trade education	24 (52.2)	18 (34.0)	8 (38.1)
University	12 (26.1)	16 (30.2)	6 (28.6)
General health			
Hypertension, n (%)	25 (54.4)	27 (50.9)	10 (47.6)
Diabetes, n (%)	6 (13.0)	12 (22.6)	3 (14.3)
Cardiovascular disease, n (%)	13 (28.3)	16 (30.2)	3 (14.3)
Stroke, n (%)	1 (2.2)	2 (3.8)	0 (0.0)
OSA symptoms			
Snoring¶, n (%)	37 (80.4)	41 (77.4)	18 (85.7)
Feeling sleepy**, n (%)	9 (20.0)	8 (15.1)	1 (4.8)
ESS score, mean±SD	6.5±3.3	6.9±4.6	6.1±4.1
ESS score>10, n (%)	4 (9.3)	10 (18.9)	4 (19.1)
Sleep study			
AHI, mean±SD	28.6±12.6	31.3±12.5	33.2±12.6
ODI, mean±SD	28.6±12.6	30.6±11.5	32.9±12.2

P values were calculated using χ² tests for categorical variables and t-tests for continuous variables, comparing long-term PAP users or adherent PAP users with non-PAP users. Statistically significant results (p<0.05) are shown in bold.

* PAP use by self-report at 2-year follow-up.

† Adherent PAP use defined as≥4 hour/night for ≥70% of nights during the last 30 days, based on objective PAP device data at 2-year follow-up.

‡ p=0.002.

§ p=0.044.

¶ Yes vs no.

** Frequent or always vs never, rarely or sometimes.

AHI, apnoea–hypopnoea index; BMI, body mass index; ESS, Epworth Sleepiness Scale; ODI, oxygen desaturation index; OSA, obstructive sleep apnoea; PAP, positive airway pressure.

Long-term PAP users were also more likely to have a BMI>30 kg/m² compared with non-PAP users (69.8% vs 50.0%, p=0.044; table 2). Otherwise, there were no significant differences between non-PAP users and long-term or adherent PAP users with respect to sex, smoking status, educational level, self-reported general health, OSA-related symptoms or OSA severity (table 2).

In logistic regression analyses, obesity (BMI>30 kg/m²) was significantly associated with long-term PAP use (online supplemental table S3). Participants with obesity had more than twofold higher odds of long-term PAP use compared with those with BMI≤30 kg/m² (adjusted OR 2.49, 95% CI 1.07 to 5.79). Age, sex, ESS score, self-reported snoring and AHI severity were not significantly associated with long-term PAP use in either crude or adjusted analyses.

## Discussion

In this population-based sample of middle-aged and older adults (54–91 years), only 7.2% had a prior diagnosis of OSA, yet 40% of those without known OSA had moderate-to-severe disease on HSAT and 30% were referred for PAP treatment following clinical evaluation. Approximately half of those identified with OSA through screening and clinical assessment subsequently became long-term PAP users, corresponding to 14% of the general population. Together, these findings indicate substantial underdiagnosis of OSA in the community and show that population-based detection, clinical assessment and long-term PAP treatment of OSA are feasible within routine care pathways.

Strengths of this study include its population-based design within a well-characterised longitudinal cohort, acceptable participation rates and use of standardised data collection. Sleep studies were scored by a single experienced technologist using contemporary scoring criteria, ensuring consistency. Clinical assessments were done by experienced clinical doctors. Referral for and delivery of PAP treatment were performed through routine clinical care pathways, enhancing real-world applicability, although this approach may have introduced variability in treatment eligibility compared with a standardised study protocol.

This study also has several limitations. The sample size was modest, particularly for subgroup analyses of PAP adherence, limiting statistical power. We did not systematically collect data on reasons for non-initiation or discontinuation of PAP therapy, limiting insight into barriers to adherence. Finally, part of the study was conducted during the COVID-19 pandemic, which may have influenced participation and treatment adherence. We observed a modest reduction in screening participation during the restriction period, while PAP adherence was numerically lower among those initiating treatment during or after the pandemic, although this difference was not statistically significant. Follow-up was partly conducted remotely, and COVID-19 infection status was not collected.

Methodological differences should be considered when comparing prevalence estimates across studies. In this study, HSAT was performed using a type 3 device without electroencephalography, which may overestimate sleep time and miss significant hypopneas that are associated with arousal but not oxygen desaturation, potentially leading to lower AHI estimates compared with full polysomnography.^1^ We used the 2012 American Academy of Sleep Medicine criteria with 3% oxygen desaturation for hypopnea instead of 4% as in older studies^12^ which is known to yield higher AHI values than the 4% criterion used in earlier studies, although this difference diminishes with increasing age.^13^ Despite these methodological considerations, the prevalence of moderate-to-severe OSA observed in this study was 45.4% among men and 33.3% among females, that is comparable to recent population-based studies like the HypnoLaus study.^14^ Notably, the HypnoLaus population was younger (mean age 57 years vs 66 years in this present study), and feasibility of clinical evaluation and offering and sustaining OSA treatment as in routine care was not evaluated. In our study, adherence to PAP therapy among individuals diagnosed through screening was comparable to that seen in routine clinical settings,^15^ supporting the feasibility of treatment in this context.

These findings have implications for clinical practice and policy. Based on our results, population-based detection of OSA using HSAT, subsequent clinical evaluation and PAP treatment initiation are technically feasible in adults, particularly those under 65 years of age. General population-based screening for OSA could therefore be considered given the 10 Principles of Screening for Diseases set by Wilson and Jungner presented already in the year 1968.^16^ However, current evidence does not support universal population screening for OSA. The US Preventive Services Task Force concluded in 2022 that evidence is insufficient to determine whether screening improves health outcomes.^17^ While PAP therapy improves patient-centred outcomes including daytime sleepiness and quality of life,^10^ randomised trials have not demonstrated clear reductions in mortality or most cardiovascular outcomes.^18^ Treatment does produce modest blood pressure reductions (2–3 mm Hg) and may reduce perioperative respiratory complications when used perioperatively.^2 10^ Our findings suggest that targeted case-finding in high-risk populations (younger adults with obesity) may be more appropriate than universal screening, though this requires validation in prospective trials designed to assess health outcomes rather than treatment uptake. Further studies are needed to define optimal age and obesity thresholds for targeted OSA screening. Importantly, our study was not designed to evaluate costs or cost-effectiveness, and therefore conclusions regarding the economic value of screening cannot be drawn.

Although PAP remains first-line treatment, alternative and more individualised approaches may improve long-term adherence. Mandibular advancement devices provide similar patient-centred benefits with higher adherence in mild to moderate OSA.^19^ Weight-reducing pharmacotherapies, including GLP-1 and GLP-1/GIP receptor agonists, also reduce AHI and represent promising options for obesity-related OSA, but long-term cardiovascular outcomes remain uncertain.^20^ Together, these developments support a shift towards more phenotype-directed treatment strategies based on symptom burden, obesity, upper airway anatomy and patient preferences.

In conclusion, our findings demonstrate that moderate-to-severe OSA is highly prevalent in a randomly selected population sample and remains substantially underdiagnosed. Participation rates were high, supporting the representativeness of the cohort and the generalisability of the findings to similar Western populations. Importantly, our results show that population-based detection of OSA using HSAT, followed by clinical evaluation and initiation of PAP therapy, is feasible within routine care pathways. Despite the challenges posed by the COVID-19 pandemic, a considerable proportion of individuals identified with OSA became long-term users of PAP therapy. Future studies focusing on larger, more targeted populations—particularly middle-aged individuals at higher risk—and incorporating more individualised treatment approaches may further improve treatment uptake and long-term adherence. In addition, formal health economic analyses are needed to assess whether the benefits of screening justify the associated costs.

## Supplementary material

10.1136/bmjopen-2026-119621 online supplemental file 1

## Data Availability

Data are available upon reasonable request.
