# Supplementary material for: Screening for obstructive sleep apnoea in the general population in Iceland and uptake of positive airway pressure treatment: a prospective cohort study
Source: BMJ Open. 2026 Sep 2;16(9):e119621. doi: 10.1136/bmjopen-2026-119621 (PMC13548229; doi:10.1136/bmjopen-2026-119621)
Supplement: Supplementary file 1 [file bmjopen-2026-119621.reviewer_comments.pdf]

PEER REVIEW HISTORY

BMJ Open publishes all reviews undertaken for accepted manuscripts. Reviewers are asked to complete a checklist review form and are provided with free text boxes to elaborate on their assessment. These free text comments are reproduced below.

ARTICLE DETAILS

Title (Provisional)

Screening for obstructive sleep apnoea in the general population in Iceland and uptake of positive airway pressure treatment: a prospective cohort study

Authors

Thorarinsdottir, Elin Helga; Benediktsdóttir, Bryndís; Aspelund, Thor; Palsdottir, Hjordis Sigrun; Hreggvidsdottir, Sesselja; Guðmundsson, Tryggvi Leó Leo; Martin, Ali Reza; Eysteinsdottir, Bjorg; Janson, Christer; Lindberg, Eva; Potts, James; Amaral, Andre F S; Gislason, Thorarinn

---

VERSION 1 - REVIEW

**Reviewer**	**1**
**Name**	**Turnbull, Christopher**
**Affiliation**	**University of Oxford, NIHR Biomedical Research Centre Oxford**
**Date**	**07-Apr-2026**
**COI**	**None**

Thorarinsdottir and colleagues present a manuscript reporting the uptake of screening for obstructive sleep apnea in middle-aged to older adults, along with long-term CPAP adherence rates in those found to have moderate to severe obstructive sleep apnea. There manuscripts shows a very high prevalence rate of OSA, confirming the findings of Benjafield and the HypnoLAUS study, whilst using home sleep testing equipment. They go on to show similar long-term adherence levels with CPAP as others have founded in clinical cohorts, rather than in the settings of screening. The low levels of long-term adherent CPAP usage at less than 25%, suggests that screening may not be a helpful approach. Nevertheless this is an important study that adds to our understanding. I have the following specific comments for the authors:

1. Participants in BOLD II were people who had participated in the BOLD I study 10 years prior. This is likely not to be a random sample and it might be that those responding were enriched for OSA, i.e. those with snoring and sleepiness might be more likely to respond. Do the authors have any information on differences between responders and non-repsonders?

2. The statistical section does not detail a linear regression analysis. Was this or any other analysis adjusted for potential confounders?

3. Only 18 participants declined or failed screening HSATs. This represents an impressively low failure rate. Can the authors divide this into those who declined or failed testing, and provide information on how many patients had to repeat a sleep study?

4. 44% of the sample had mild OSA which can respond to CPAP or other therapies when it has a marked impact on QOL. How were these participants treated?

5. Section 3.3.1 details factors associated with CPAP adherence. Are factors considered in univariate analysis or is this a multivariate model?

6. Only 2 older participants were CPAP adherent in the longer-term. It might be possible that the financial barrier of paying for therapy was greater in this group. Unfortunately there is no qualitative analysis or patient/ public input to understand what the barriers might have been to therapy.

7. The impact of screening on health economics is not known limiting the conclusions that can be drawn in terms of the cost-efficacy of screening.

Reviewer **2**

Name **Bussadori, Sandra**

Affiliation **Universidade Nove de Julho - Campus Vergueiro, Postgraduation Program in Medicine Biophotonics**

Date **23-Apr-2026**

COI **None**

I would like to congratulate you on the conduct of this study and the overall robustness of the methodology. I have a few minor suggestions that may help strengthen the manuscript.

* Part of the study appears to have been conducted during the COVID-19 pandemic, including a shift to remote follow-up. It would be helpful if the authors could clarify whether this period may have influenced PAP adherence and sleep-related outcomes. Were any analyses performed to account for potential differences between pre-pandemic and pandemic periods? Additionally, please indicate whether COVID-19 infection status was collected and, if so, whether it was considered in the analyses. A brief discussion of these aspects would strengthen the manuscript.

* A STROBE checklist was provided; it would be useful to explicitly state in the manuscript that reporting follows STROBE recommendations.

* In the Discussion section, I suggest including a clearer and more structured description of the study limitations.

* The manuscript could benefit from a brief perspective on emerging approaches in sleep apnea management, with appropriate contextualization of newer modalities such as laser-based interventions.

* The final paragraph of the Discussion would benefit from clearer structure and language revision. While important points are raised, it would benefit from a more explicit concluding statement that clearly synthesizes the main findings and their clinical implications.

VERSION 1 - AUTHOR RESPONSE

Reviewers Comments to the Author:

Reviewer 1

Comments to the Author:

Thorarinsdottir and colleagues present a manuscript reporting the uptake of screening for obstructive sleep apnea in middle-aged to older adults, along with long-term CPAP adherence rates in those found to have moderate to severe obstructive sleep apnea. There manuscripts shows a very high prevalence rate of OSA, confirming the findings of Benjafield and the HypnoLAUS study, whilst using home sleep testing equipment. They go on to show similar long-term adherence levels with CPAP as others have founded in clinical cohorts, rather than in the settings of screening. The low levels of long-term adherent CPAP usage at less than 25%, suggests that screening may not be a helpful approach. Nevertheless this is an important study that adds to our understanding. I have the following specific comments for the authors:

Comment 1.1: Participants in BOLD II were people who had participated in the BOLD I study 10 years prior. This is likely not to be a random sample and it might be that those responding were enriched for OSA, i.e. those with snoring and sleepiness might be more likely to respond. Do the authors have any information on differences between responders and non-repsonders?

Response 1.1:

We thank the reviewer for this important comment regarding potential selection bias in the BOLD II follow-up cohort.

The baseline Icelandic BOLD I study was based on a random general population sample of Icelanders 40 years and older and had a high participation rate. We have previously shown

that the responders were broadly representative of the underlying population, with no significant differences between responders and non-responders in terms of age, smoking status, or general health, although a slightly higher proportion of men participated ($p=0.01$) (Thorleifsson et al., doi: 10.1016/j.rmed.2009.04.005)

Loss to follow-up over the 10-year period was primarily driven by ineligibility, including mortality (n=187 in the Icelandic cohort), rather than selective non-response (Amaral et al., doi: 10.1093/ije/dyad146)

Importantly, the BOLD study was designed to investigate obstructive lung disease, and sleep-related assessments were only briefly introduced as such to participants at BOLD II. We therefore, find it unlikely that participation in BOLD II has been specifically driven by OSA symptoms such as snoring or sleepiness.

To further address the reviewer's concern, that the BOLD II responders were enriched for obstructive sleep apnea we compared baseline characteristics in BOLD I between those participants who took part in BOLD II (responders) and those who did not (non-responders). Given the long follow-up time between BOLD I and II the BOLD II responders were younger than non-responders (mean age 51.7 vs 61.0 years, $p<0.001$). There were no significant differences in BMI (27.9 vs 28.0 kg/m^2, $p=0.73$) or frequency of habitual snoring (45.0% vs 47.1%, $p=0.57$). BOLD II responders had slightly higher Epworth Sleepiness Scale scores (6.7 vs 5.3, $p<0.001$) in BOLD I. Excessive daytime sleepiness (Epworth Sleepiness Scale score >10) was somewhat higher among responders but the difference did not reach statistical significance (57.8% vs 42.2%, $p=0.061$).

These results have now been incorporated into the manuscript (see results section 3.1, page 8, lines 25-31).

Comment 1.2: The statistical section does not detail a linear regression analysis. Was this or any other analysis adjusted for potential confounders?

Response 1.2: We thank the reviewer for this important comment. In the original version of the manuscript, we had not performed regression analyses adjusted for potential confounders. We have now expanded the statistical analyses to include linear regression analyses and

multivariable regression analyses evaluating factors associated with AHI severity and long-term PAP use. These analyses were adjusted for relevant potential confounders. For the analyses exploring factors associated with long-term PAP use, adjustment was limited to age, sex, and BMI due to the relatively small sample size (n=99). Overall, the adjusted analyses were largely consistent with the univariate findings. The statistical methods section has been updated accordingly (page 7, lines 33-34, page 8, lines 1-4), and the results are now described in the Results section and Supplementary Tables S1–S3 (page 10 lines 4-10 and page 11 lines 22-26).

Comment 1.3: Only 18 participants declined or failed screening HSATs. This represents an impressively low failure rate. Can the authors divide this into those who declined or failed testing, and provide information on how many patients had to repeat a sleep study?

Response 1.3:
In response to this comment, we have clarified this in more detail. Among the 378 participants, 26 had a prior OSA diagnosis and and 2 had undergone recent OSA screening (<1 year) that showed no OSA and were therefore not re-screened and not included in the current analysis. Twelve declined screening. Altogether 35 HAST studies had to be repeated due to various reasons. All but 4 had eventually returned with an acceptable HAST while 4 declined repeated testing. Thus 334 accepted and completed a technically acceptable HAST screening.
We have now added this more detailed information in figure 1 and in results section 3.1 (page 9, lines 1-6)

Comment 1.4: 44% of the sample had mild OSA which can respond to CPAP or other therapies when it has a marked impact on QOL. How were these participants treated?

Response 1.4: We appreciate this important comment.
All participants received written information regarding their clinical results, including findings from the sleep study, spirometry, and blood tests, to support further clinical assessment and management as appropriate. Participants with mild OSA were informed of their diagnosis and advised to seek further evaluation and management from their general practitioner. In addition, a summary of the results was sent to each participant's general practitioner and other treating physicians when applicable.

This has now been clarified in the Methods section (section 2.2.4, page 6, lines 25-29).

Comment 1.5: Section 3.3.1 details factors associated with CPAP adherence. Are factors considered in univariate analysis or is this a multivariate model?

Response 1.5: We thank the reviewer for this important comment. The analyses originally presented in Section 3.3.1 were based on univariate comparisons (t-tests and χ^2 tests). To further address potential confounding, we have now added logistic regression and multivariable logistic regression analyses evaluating factors associated with long-term PAP use. These additional analyses are now presented in Supplementary Table S3 and described in the Results section (page 11, lines 22-26). Overall, the adjusted analyses were consistent with the univariate findings, with obesity remaining significantly associated with long-term PAP use.

Comment 1.6: Only 2 older participants were CPAP adherent in the longer-term. It might be possible that the financial barrier of paying for therapy was greater in this group. Unfortunately there is no qualitative analysis or patient/ public input to understand what the barriers might have been to therapy.

Response 1.6: We thank the reviewer for highlighting this point. We would like to clarify that we previously misstated the reduced PAP user fee for elderly individuals. The reduced fee is approximately USD 11 every three months (rather than per month), corresponding to approximately USD 4 monthly. We have corrected this in the manuscript (see page 6, line 33). Given this relatively modest cost, we believe financial burden is unlikely to have substantially influenced PAP adherence in this elderly cohort. However, we did not systematically collect data regarding reasons for non-adherence and are therefore unable to directly assess the role of financial or other barriers. This is now mentioned as a limitation in the discussion (see page 12, lines 11-13).

Comment 1.7: The impact of screening on health economics is not known limiting the conclusions that can be drawn in terms of the cost-efficacy of screening.

Response 1.7: We agree with the reviewer that our study was not designed to assess the health economic impact of population-based OSA screening. We also emphasize that the primary aim of this study was to evaluate the feasibility of population-based OSA detection,

clinical assessment, and PAP treatment within routine care pathways. We have now clarified in the discussion that conclusions regarding the cost-effectiveness of screening cannot be drawn from the present study (page 13, lines 16-18).

Reviewer: 2

Comments to the Author:

I would like to congratulate you on the conduct of this study and the overall robustness of the methodology. I have a few minor suggestions that may help strengthen the manuscript.

Response: Thank you very much for these kind and encouraging words.

Comment 2.1: Part of the study appears to have been conducted during the COVID-19 pandemic, including a shift to remote follow-up. It would be helpful if the authors could clarify whether this period may have influenced PAP adherence and sleep-related outcomes. Were any analyses performed to account for potential differences between pre-pandemic and pandemic periods? Additionally, please indicate whether COVID-19 infection status was collected and, if so, whether it was considered in the analyses. A brief discussion of these aspects would strengthen the manuscript

Response 2.1: We thank the reviewer for this important comment. The study period overlapped with the COVID-19 pandemic in Iceland, during which public health restrictions were implemented from 16 March 2020 to end of February 2022. To further clarify the temporal relationship between study procedures and the COVID-19 restriction period, we provide a schematic timeline below for the reviewer's reference.

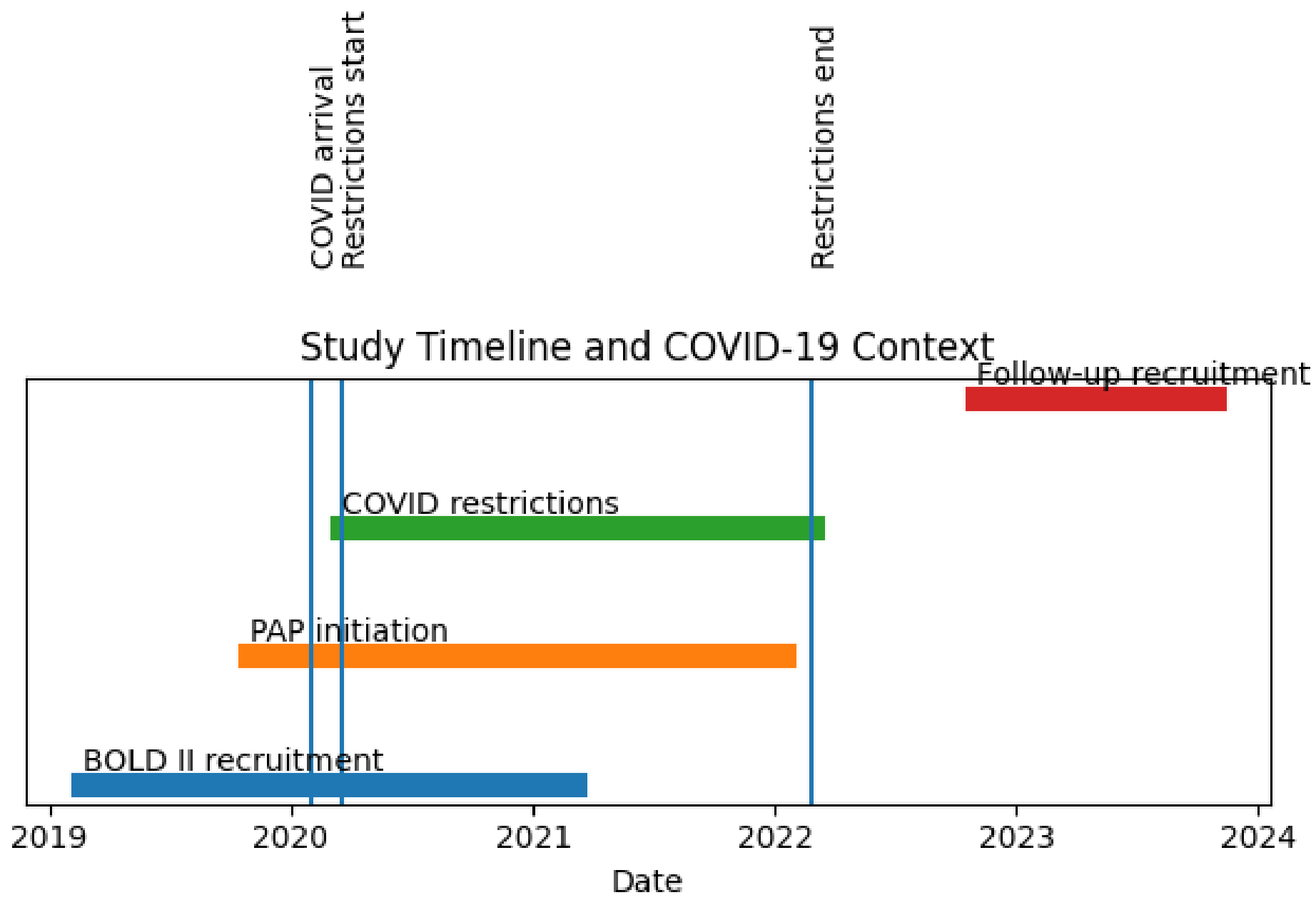

To assess the potential impact of COVID-19 during this period, we have now performed additional analyses. In total, 256 participants were recruited in the BOLD II study before COVID-19 restrictions on 16 March 2020 and 122 during the restriction period. Participation in OSA screening was significantly lower during the restriction period compared with the pre-pandemic period (90.8% vs 97.1%, p=0.012).

We have also examined PAP adherence according to timing of PAP treatment initiation. Only six participants did initiate PAP therapy before the COVID-19 restrictions. Adherence at the 2-year follow-up was numerically lower among participants who initiated PAP therapy during the COVID-19 period compared with those who started before (53.3% vs 83.3%), although this difference was not statistically significant (p=0.152). These findings should be interpreted with caution due to the small number of participants initiating treatment before the pandemic (n=6). In addition, individuals with more severe OSA may have been prioritized for earlier treatment, as reflected by a somewhat higher mean AHI among those who initiated treatment before the pandemic compared with those who started later (37.7 ± 17.9 vs 29.8 ± 12.2, p=0.139).

COVID-19 infection status was not collected as part of this study, and therefore could not be included in the analyses. During periods of high COVID-19 prevalence, in-person clinical

services were totally stopped and follow-up was only conducted per telephone, which may have influenced treatment adherence.

These irregularities in patient care during COVID-19 have now been clarified in the methods (page 5, line 4-7, results, page 8, lines 4-9), results (page 10, lines 31-33 and page 11 lines 1-2) and discussion (page 12, lines 13-18).

Comment 2.2: A STROBE checklist was provided; it would be useful to explicitly state in the manuscript that reporting follows STROBE recommendations.

Response 2.2: We thank the reviewer for this suggestion. We have now added a statement in the end of methods section indicating that the study is reported in accordance with STROBE guidelines (see page 8, lines 12-13).

Comment 2.3: In the Discussion section, I suggest including a clearer and more structured description of the study limitations.

Response 2.3: We appreciate this helpful suggestion. We have revised the Discussion section to provide a clearer and more structured description of the study limitations. In particular, the limitations are now presented more systematically, including aspects related to sample size, measurement methods, treatment delivery within routine clinical care, the potential impact of the COVID-19 pandemic and the lack of systematic evaluation of non-compliance to PAP (see page 12, lines 10-18)

Comment 2.4: The manuscript could benefit from a brief perspective on emerging approaches in sleep apnea management, with appropriate contextualization of newer modalities such as laser-based interventions.

Response 2.4: We have expanded the discussion to include a brief perspective on emerging approaches in the management of OSA (see page 13, lines 24-26). We did consider adding further references but we have already reached the maximum of 20 and to cover the laser-based interventions would have needed considerable space given the latest review and meta-analysis (see reference doi: 10.1093/sleep/zsx004).

Comment 2.5: The final paragraph of the Discussion would benefit from clearer structure and language revision. While important points are raised, it would benefit from a more

explicit concluding statement that clearly synthesizes the main findings and their clinical implications.

Response 2.5: We thank the reviewer for this helpful suggestion. We have revised the final paragraph of the discussion to improve clarity and structure and to include a more explicit concluding statement (see page 13, lines 27-32 and page 14 lines 1-6)

VERSION 2 - REVIEW

**Reviewer**	**1**
**Name**	**Turnbull, Christopher**
**Affiliation**	**University of Oxford, NIHR Biomedical Research Centre Oxford**
**Date**	**25-Jun-2026**
**COI**	

Many for your comprehensive response to my previous comments. I have no further comments or concerns.
