# Supplementary material for: Screening for obstructive sleep apnoea in the general population in Iceland and uptake of positive airway pressure treatment: a prospective cohort study
Source: BMJ Open. 2026 Sep 2;16(9):e119621. doi: 10.1136/bmjopen-2026-119621 (PMC13548229; doi:10.1136/bmjopen-2026-119621)
Supplement: Supplementary file 2 [file bmjopen-16-9-s001.docx]

**Table S1.** Factors associated with moderate OSA (AHI≥15)

| Variable | Crude OR (95% CI) | Adjusted OR (95% CI) | Crude  p-value | Adjusted p-value |
| --- | --- | --- | --- | --- |
| Age (per 10-year increase) | 1.47 (1.12 – 1.94) | 1.75 (1.24 – 2.47) | **0.005** | **0.001** |
| Male gender | 1.70 (1.06 – 2.59) | 1.90 (1.10 – 3.27) | **0.026** | **0.021** |
| BMI category (kg/m^2^) |  |  |  |  |
| 25-29.9 (vs <25) | 2.3 (1.10 – 4.80) | 1.79 (0.78 – 4.09) | **0.026** | 0.169 |
| ≥30 (vs <25) | 7.98 (3.80 – 16.78) | 6.43 (2.77 – 14.88) | **<0.001** | **<0.001** |
| ESS score (>10 vs ≤10) | 0.64 (0.35 – 1.18) | 0.53 (0.26 – 1.10) | 0.151 | 0.087 |
| Snoring (yes vs no) | 2.4 (1.30 – 4.43) | 2.71 (1.32 – 5.56) | **0.005** | **0.006** |

Crude ORs derived from logistic regression. Adjusted ORs derived from multivariable logistic regression including age, sex, BMI category, ESS score, and snoring.

Abbreviations: AHI = Apnea Hypopnea Index, OR = Odds ratio, 95% CI = 95% Confidence interval, BMI = Body Mass Index, ESS = Epworth Sleepiness Scale

**Table S2.** Characteristics of the 132 participants with AHI≥15 on screening, comparing subjects that were referred and not referred for PAP treatment.

|  | Subjects with AHI ≥15 | Subjects referred for PAP treatment | Subjects not referred for PAP treatment | p-value* |
| --- | --- | --- | --- | --- |
| Subjects, n | 132 | 99 | 24 | - |
| Demographics |  |  |  |  |
| Male gender, n (%) | 80 (60.6) | 59 (59.6) | 16 (66.7) | 0.524 |
| Age, years ± SD | 67.9 ± 8.8 | 67.2 ± 8.6 | 71.0 ± 9.0 | 0.053 |
| Mean BMI, kg/m2 ± SD | 31.5 ± 5.9 | 31.4 ± 5.1 | 31.6 ± 9.0 | 0.865 |
| BMI >30 kg/m2, n (%) | 76 (57.6) | 60 (60.6) | 9 (37.5) | **0.041** |
| Smoking, n (%) |  |  |  | 0.686 |
| Current | 4 (3.0) | 3 (3.0) | 0 (0.0) |  |
| Former | 74 (56.1) | 57 (57.6) | 14 (58.3) |  |
| Never | 54 (40.9) | 39 (39.4) | 10 (41.7) |  |
| Highest level of education, n (%) |  |  |  | 0.655 |
| None | 0 (0.0) | 0 (0.0) | 0 (0.0) |  |
| Primary school | 5 (3.8) | 4 (4.0) | 1 (4.2) |  |
| Lower secondary school | 17 (12.9) | 13 (13.1) | 2 (8.3) |  |
| Upper secondary school | 15 (11.4) | 12 (12.1) | 3 (12.5) |  |
| Vocational or trade education | 60 (44.5) | 42 (42.4) | 14 (58.3) |  |
| University | 35 (26.5) | 28 (28.3) | 4 (16.7) |  |
| General health |  |  |  |  |
| Hypertension, n (%) | 68 (51.5) | 52 (52.5) | 12 (50.0) | 0.824 |
| Diabetes, n (%) | 20 (15.2) | 18 (18.2) | 2 (8.3) | 0.241 |
| Cardiovascular disease, n (%) | 33 (25.0) | 29 (29.3) | 4 (16.7) | 0.210 |
| Stroke, n (%) | 3 (3.0) | 3 (3.1) | 0 (0.0) | 0.385 |
| OSA symptoms |  |  |  |  |
| Snoring †, n (%) | 97 (77.0) | 78 (78.8) | 19 (66.7) | 0.209 |
| Feeling sleepy ‡, n (%) | 23 (18.6) | 17 (17.4) | 4 (17.4) | 0.996 |
| ESS score, mean ± SD | 6.7 ± 4.0 | 6.7 ± 4.1 | 6.4 ± 3.9 | 0.762 |
| ESS score >10, n (%) | 17 (13.9) | 14 (14.6) | 2 (8.7) | 0.457 |
| Sleep study |  |  |  |  |
| AHI, mean ± SD | 27.9 ± 12.0 | 30.1 ± 12.5 | 20.0 ± 4.4 | **<0.001** |
| ODI, mean ± SD | 27.6 ± 11.6 | 29.7 ± 12.0 | 20.1 ± 4.8 | **<0.001** |

*p values were calculated using χ² tests for categorical variables and t tests for continuous variables, comparing participants referred for PAP treatment with those not referred. Statistically significant results (p < 0.05) are shown in bold.
† Yes versus no.
‡ Frequent or always versus never, rarely, or sometimes.

**Abbreviations:** AHI, apnoea–hypopnoea index; ODI, oxygen desaturation index; SD, standard deviation; BMI, body mass index; ESS, Epworth Sleepiness Scale.

**Table S3**. Factors associated with long term PAP use*

| Variable | Crude OR (95% CI) | Adjusted OR (95% CI) | Crude  p-value | Adjusted p-value |
| --- | --- | --- | --- | --- |
| Age (per year) | 0.98 (0.93 – 1.03) | 0.98 (0.93 – 1.03) | 0.323 | 0.328 |
| Male gender | 1.27 (0.57 – 2.84) | 1.28 (0.54 – 3.03) | 0.562 | 0.576 |
| BMI (kg/m^2^) (>30 vs ≤30) | 2.31 (1.02 – 5.27) | 2.49 (1.07 – 5.79) | **0.046** | **0.034** |
| ESS score (>10 vs ≤10) | 2.44 (0.71 – 8.40) | 2.23 (0.63 – 7.93) | 0.157 | 0.215 |
| Snoring (yes vs no) | 1.11 (0.30 – 4.14) | 1.29 (0.29 – 5.67) | 0.879 | 0.740 |
| AHI ( per event/hour) | 1.02 (0.99 – 1.05) | 1.01 (0.98 – 1.05) | 0.290 | 0.409 |

* PAP use by self-report at 2-year follow-up

Crude ORs derived from logistic regression. Adjusted ORs derived from multivariable logistic regression adjusted for age, sex and BMI.

Abbreviations: AHI = Apnea Hypopnea Index, OR = Odds ratio, 95% CI = 95% Confidence interval, BMI = Body Mass Index, ESS = Epworth Sleepiness Scale
